# MicroRNA-22 suppresses the growth, migration and invasion of colorectal cancer cells through a Sp1 negative feedback loop

**DOI:** 10.18632/oncotarget.16742

**Published:** 2017-03-31

**Authors:** Shu-Sen Xia, Guang-Jun Zhang, Zuo-Liang Liu, Hong-Peng Tian, Yi He, Chang-Yuan Meng, Li-Fa Li, Zi-Wei Wang, Tong Zhou

**Affiliations:** ^1^ The Department of Gastrointestinal Surgery, The First Affiliated Hospital of Chongqing Medical University, Chongqing, People's Republic of China; ^2^ The Department of Gastrointestinal Surgery, The Affiliated Hospital of North Sichuan Medical College, Nanchong, Sichuan, People's Republic of China; ^3^ Institute of Hepatobiliary, Pancreatic and Intestinal Disease, North Sichuan Medical College, Nanchong, Sichuan, People's Republic of China; ^4^ The Department of Pathology, North Sichuan Medical College, Nanchong, Sichuan, People's Republic of China; ^5^ The Department of Medical Microbiology and Parasitology, North Sichuan Medical College, Nanchong, Sichuan, People's Republic of China

**Keywords:** colorectal cancer, miR-22, Sp1, PTEN, AKT

## Abstract

MicroRNAs have recently emerged as regulators of many biological processes including cell proliferation, development and differentiation. This study identified that miR-22 was statistically decreased in colorectal cancer clinical specimens and highly metastatic cell lines. Moreover, low miR-22 expression was associated with tumor metastasis, advanced clinical stage and relapse. Consistent with clinical observations, miR-22 significantly suppressed the ability of colorectal cancer cells to growth and metastasize *in vitro* and *in vivo*. Sp1 was validated as a target of miR-22, and ectopic expression of Sp1 compromised the inhibitory effects of miR-22. In addition, Sp1 repressed miR-22 transcription by binding to the miR-22 promoter, hence forming a negative feedback loop. Further study has shown that miR-22 suppresses the activity of PTEN/AKT pathway by Sp1. Our present results implicate the newly indentified miR-22/Sp1/PTEN/AKT axis might represent a potential therapeutic target for colorectal cancer.

## INTRODUCTION

In China, the incidence of colorectal cancer(CRC) is continually increasing despite advances in treatment and subsequent improvement in prognosis [1]. However, the origin and development of CRC are complex and still obscure. It has been revealed that the altered expression or activity of specific genes, including microRNAs (miRNAs), is involved in the pathogenesis of CRC [2, 3].

MiRNAs are small non-coding RNAs (~22nt) that recognize and bind to partially complementary sequences of their target mRNA, resulting in either mRNA degradation or inhibition of its translation [4, 5]. MiRNAs regulate the expression of a wide variety of target genes, and are therefore involved in a wide range of biological processes [6, 7]. Accumulating evidences show that miRNAs are often abnormally expressed in diverse cancers [8, 9]. Among the deregulated miRNAs, miR-22 is widely studied in various cancers, including CRC [10–19]. Our previous studies have also suggested that miR-22 is significantly down-regulated in CRC tissues and low expression of miR-22 correlated with distant metastases and poor prognosis of CRC [20].

Specificity protein 1 (Sp1) plays an important role in many pathophysiological processes, including cell cycle progression, angiogenesis and cell migration [21, 22]. It can affect tumor progression and metastasis by modulating the expression of its target genes [23, 24]. In CRC, activation of Sp1 often positively correlates with tumor malignancy and indicates poor prognosis [25, 26].

Given the importance of both miR-22 and Sp1 in the processes linked to CRC, the precise regulatory relationship between Sp1 and miR-22 in CRC cells remains poorly understood. In this study, we found that miR-22 expression is down-regulated in late-stage CRC and is associated with metastasis and relapse of CRC patients. In addition, miR-22 directly targeted Sp1 and thereby suppressed the activation of the PTEN-AKT pathway, and Sp1 attenuated the inhibitory effects of miR-22 in CRC cells. Conversely, Sp1 directly inhibited miR-22 expression, and miR-22 suppressed Sp1-induced CRC cell proliferation, growth, migration and invasion, implying the existence of a double-negative feedback loop between Sp1 and miR-22.

## RESULTS

### Down-regulation of miR-22 in human CRC was associated with disease progression and metastasis

**Figure 1:**
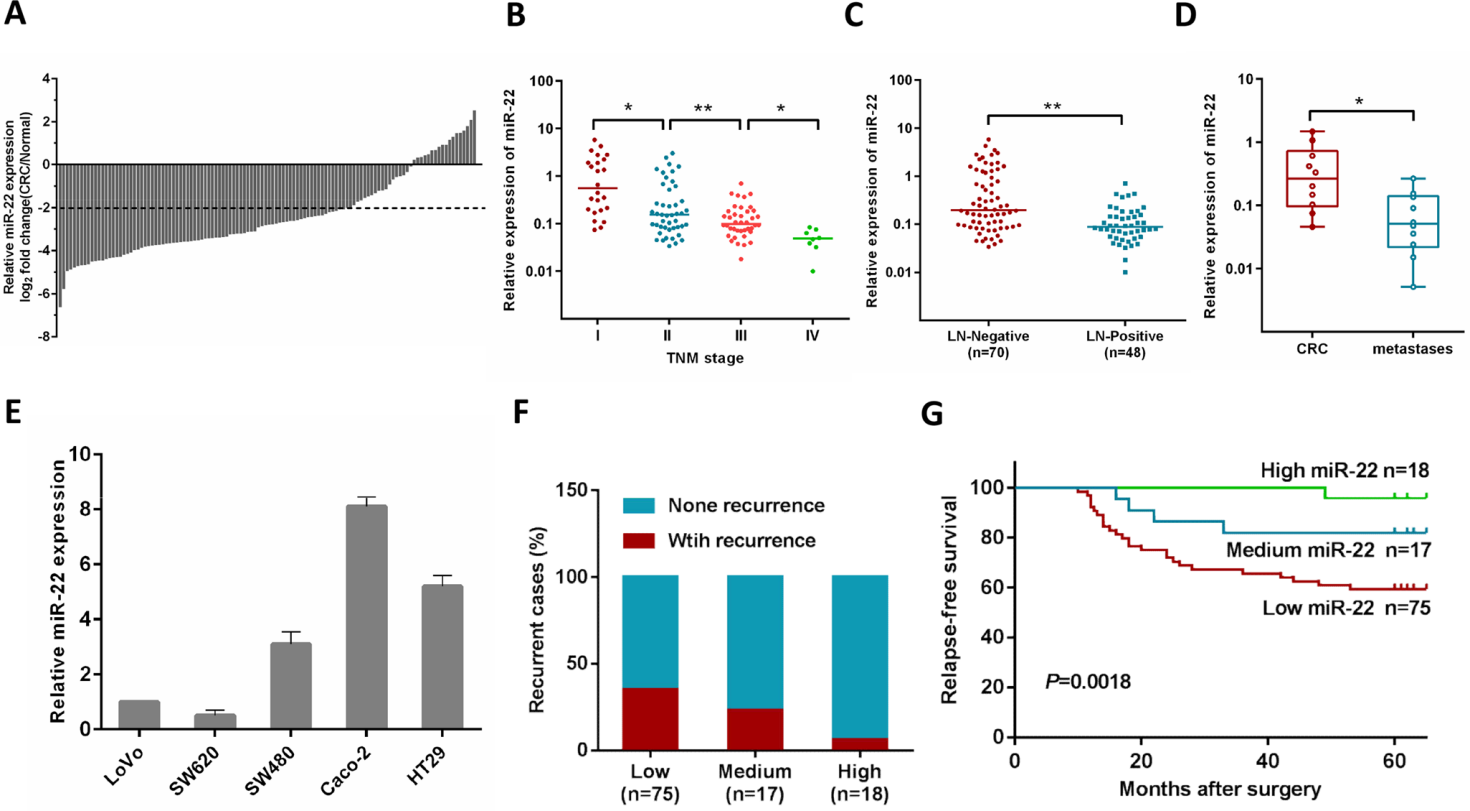
MiR-22 level is associated with CRC progression, metastasis and relapse (**A**) Comparison of miR-22 expression level between primary CRC samples and paired adjacent normal tissues. A log_2_ fold change (CRC/normal) less than −2 was considered a significant down-regulation (dotted lines). (**B**) miR-22 expression levels in different clinical stages of CRC patients. (**C**) miR-22 is differentially expressed in the lymph node metastasis negative group (LN-Negative) compared with the positive group (LN-Positive). (**D**) The relative expression of miR-22 in matched primary CRC tissues and liver metastatic tissues. (**E**) The relative expression of miR-22 in five CRC cell lines (HT29, Caco-2, SW480, SW620 and LoVo). (**F**) The correlation of miR-22 expression and CRC recurrence was analyzed. (**G**) Kaplan-Meier analysis of relapses-free survival of Stage I–III CRC patients by miR-22 expression. *P < 0.05, **P < 0.01.

Our previous study showed that the expression of miR-22 was significantly lower in CRC tissues, and low expression of miR-22 was correlated with liver metastasis [20]. In the present study, we investigated the miR-22 expression level in an expanded CRC cohort consisting of 118 pairs of primary CRC and their matched normal tissues. 76% (83 of 118) of the CRC patients had reduced miR-22 expression by at least 4-fold as compared with their matched normal tissues (Figure 1A), and miR-22 correlated with tumor progression as it was decreased in the sequence from stage I to stage IV CRC (Figure 1B). In addition, we found that the miR-22 expression levels were significantly down-regulated in the tissues of CRC patients with lymph node metastasis compared with those without lymph node metastasis (Figure 1C).

We then analyzed miR-22 expression in primary tumors and liver metastatic tissues from 10 patients with metastatic CRC, and found that miR-22 expression was lower in the metastases than their primary tumors (Figure 1D). Consistent with these observations, miR-22 levels were significantly lower in metastatic CRC cell lines (SW620 and Lovo), compared with non-metastatic ones (SW480, HT29 and Caco-2) (Figure 1E).

### Correlation between miR-22 expression and relapse-free survival of colorectal cancer patients

In this study, postoperative recurrence was observed in 28.2% cases (31/110) among 110 patients with stage I–III disease who underwent curative resection. To test if miR-22 is associated with relapse-free survival in CRC, the patients were stratified according to the logarithmic ratio between miR-22 expression in cancer and normal tissues: high (log2 miR-22 expression > 0), medium (0 < log2 miR-22 expression < −2), and low (log2 miR-22 expression < −2). Clinical investigations showed that the patients with low miR-22 levels possessed a higher risk of CRC recurrence rate (*P* = 0.043, Figure 1F). Using Kaplan-Meier analysis, the results also demonstrated that CRC patients with low miR-22 levels displayed a higher recurrence rate than patients with high miR-22 expression (Figure 1G). Furthermore, Cox multivariate analysis was performed to identify independent prognostic markers for relapse-free survival. Since TNM stage is determined by tumor depth and lymph node metastasis, it was not further enrolled into the multivariate analysis in this study. The results confirmed that miR-22 expression and lymph node metastasis were independent prognostic factors, indicating that miR-22 could be used as a biomarker of early recurrence of CRC (Supplementary Table 3).

### MiR-22 suppresses CRC cell proliferation, colony formation, migration and invasion abilities *in vitro*

**Figure 2:**
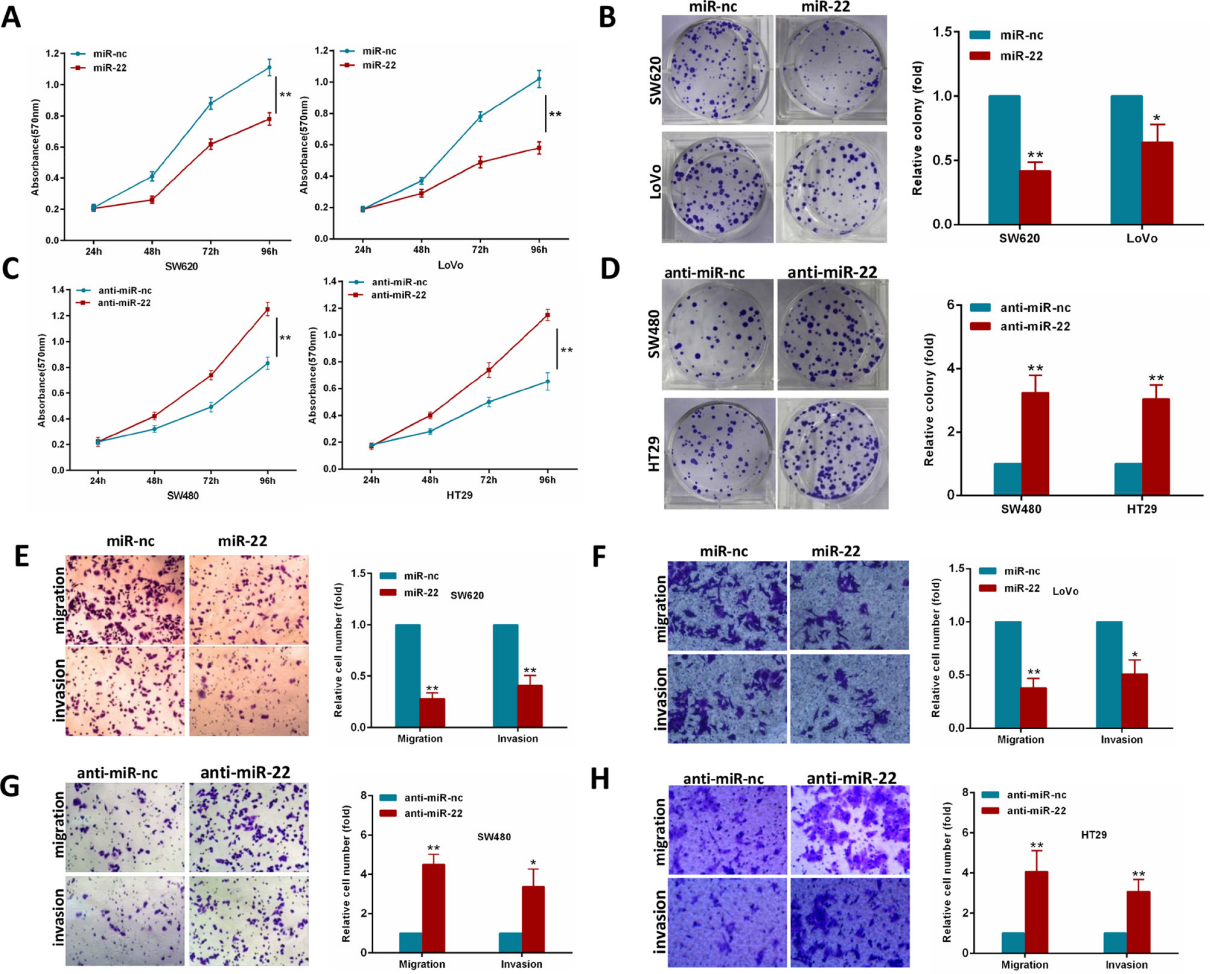
MiR-22 modulates CRC cell proliferation, colony formation, migration and invasion (**A**, **B**) MTT and colony formation assays were performed in SW620 and LoVo cells expressing miR-22 or the control. (**C**, **D**) MTT and colony formation assays were performed in SW480 and HT29 cells transiently transfected with miR-22 inhibitor or with control inhibitor. (**E**, **F**) SW620 and LoVo cells transfected with miR-22 mimics or with control mimics were subjected to transwell migration and invasion assays. (**G**, **H**) SW480 and HT29 cells transfected with miR-22 inhibitor or with control inhibitor were subjected to transwell migration and invasion assays. *P < 0.05, **P < 0.01.

To explore whether miR-22 suppresses an oncogenic phenotype of CRC, we performed gain and loss function assays in CRC cells by using a miR-22 mimics or inhibitor. Overexpression of miR-22 resulted in a significant decrease in proliferation and colony formation in SW620 and LoVo cells (Figure 2A, 2B). In contrast, as shown in Figure 2C, 2D, miR-22 inhibition significantly increased proliferation and colony formation compared with the scramble controls in SW480 and HT29 cells. Furthermore, we performed transwell migration and invasion assays to determine whether miR-22 regulates CRC cell migration and invasion abilities. We found that miR-22 suppressed the migration and invasion of SW620 and LoVo cells (Figure 2E, 2F). Conversely, miR-22 inhibition significantly promoted SW480 and HT29 cell migration and invasion (Figure 2G, 2H). These observations demonstrate that miR-22 significantly suppresses proliferation, colony formation, migration and invasion of CRC cells.

### MiR-22 inhibits CRC growth and metastasis *in vivo*

**Figure 3:**
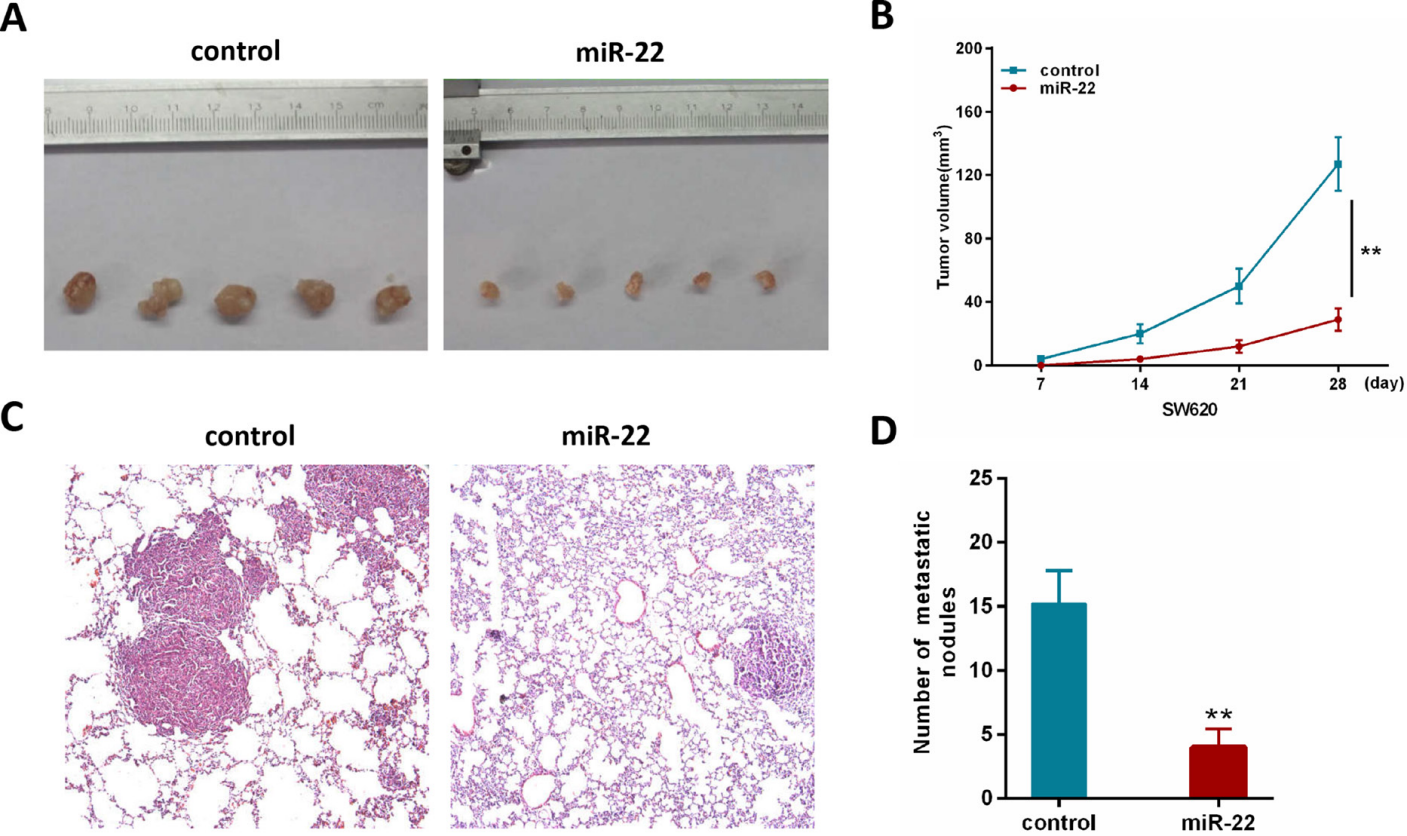
MiR-22 suppresses the growth and metastatic ability of CRC cells *in vivo* (**A**) Effects of miR-22 on subcutaneous tumor generation. (**B**) Tumor sizes were measured on the indicated days (d). (**C**) Representative H&E staining images using a dissection microscope showed metastatic lesions in the lungs of mice injected with miR-22 overexpressing SW620 cells or control cells (×100 magnification). (**D**) The number of lung metastatic nodules per mouse was counted under the microscope.

To further investigate the contribution of miR-22 *in vivo*, we constructed a SW620 cell line stably overexpressing miR-22 by retroviral-mediated transfection. Firstly, we subcutaneously injected SW620/miR-22 and control cells into nude mice. The subcutaneous tumor volume in miR-22 group was significantly decreased compared with control group (Figure 3A, 3B). Secondly, we conducted an experimental metastasis assay by injecting SW620/miR-22 and control cells into the tail vein of nude mice, and quantified the number of metastatic nodes formed in the lungs after 8 weeks. As shown in Figure 3C, large lung metastatic nodules could be detected in control group, while only few small nodules were observed in miR-22 group. Moreover, the number of metastatic nodules was obviously reduced in the group injected with SW620/miR-22 cells (Figure 3D). These observations are consistent with our *in vitro* results and add further evidence to miR-22's role as a tumor suppressor in CRC.

### Sp1 is a direct target of miR-22

**Figure 4:**
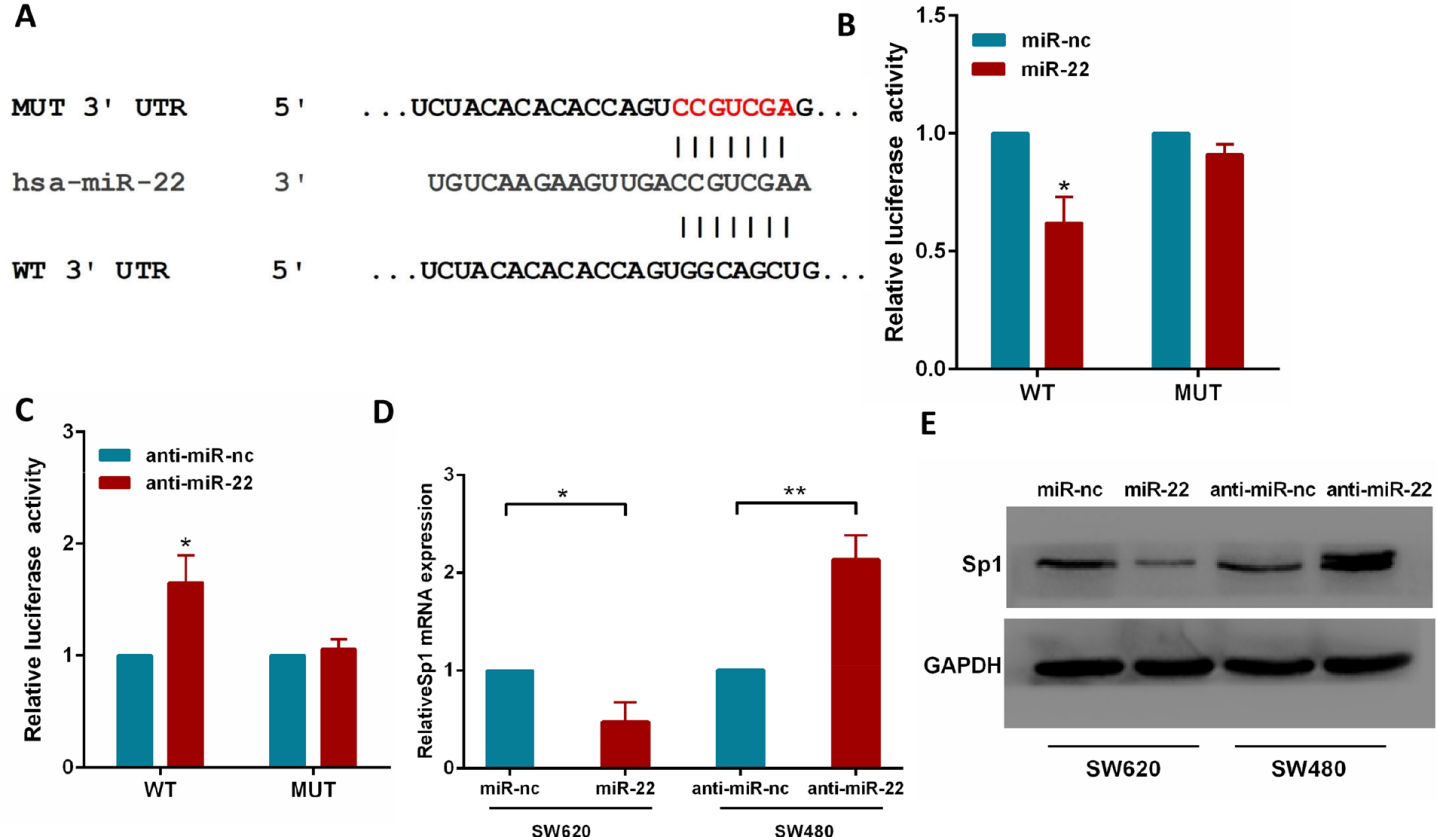
Sp1 is a direct target of miR-22 (**A**) The wild-type and mutant of putative miR-22 target sequences of Sp1 3′UTR. (**B**, **C**) Analysis of the luciferase activity of psicheck-2-Sp1 3′UTR WT and MUT vector in HEK293T cells by miR-22 or anti-miR-22. (**D**) The Sp1 mRNA levels in the indicated cells was analyzed by qRT-PCR. (**E**) The Sp1 protein levels in the indicated cells were examined by western blot. *P < 0.05, **P < 0.01.

To fully understand the mechanisms by which miR-22 executed its function, we adopted the bioinformatic algorithms (MiRanda, TargetScan, and PicTar) for target gene prediction. Among these candidates, Sp1 was identified as a potential target of miR-22 and selected for further analysis, as it is an important oncogene which is up-regulated in several tumor types [27–29]. The predicted binding site of miR-22 was observed in the 3′UTR of Sp1 mRNA (Figure 4A).

To test the function of this potential binding site, we inserted wild-type or mutant 3′UTR sequences immediately downstream of the luciferase reporter gene and co-expressed these with either miR-22 or anti-miR-22 in HEK293T cells. As shown in Figure 4B, 4C, miR-22 overexpression caused a significant decrease in relative luciferase activity, whereas miR-22 silencing increased the luciferase activity. In addition, mutation of the binding site of miR-22 in the 3′UTR of Sp1 abolished both the effect of miR-22 and anti-miR-22 (Figure 4B, 4C), confirming that miR-22 can bind to the Sp1 3′UTR. Furthermore, qRT-PCR and western blotting analyses showed that miR-22 overexpression significantly reduced the levels of Sp1 mRNA and protein in SW620 cells, while miR-22 knockdown increased Sp1 levels (Figure 4D, 4E). Together, these results strongly support a direct suppression of Sp1 by miR-22 by means of mRNA degradation as well as translational repression.

### miR-22 suppresses CRC cell growth and invasion through Sp1

**Figure 5:**
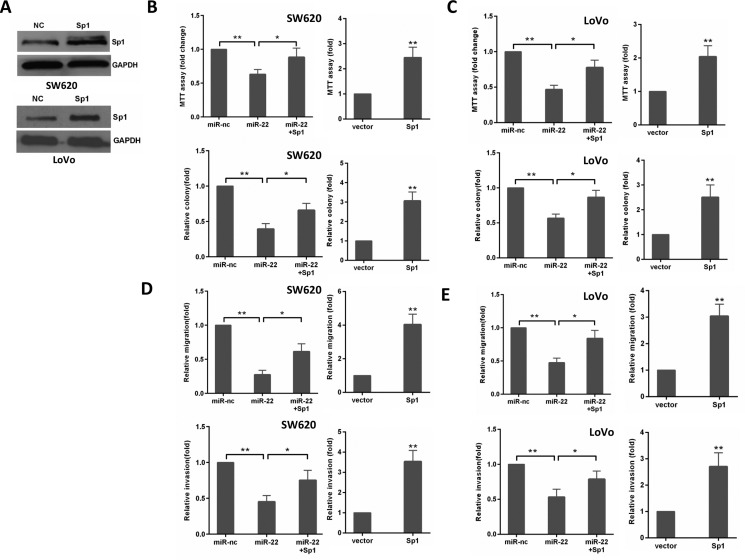
The levels of Sp1 influence the effects of miR-22 in CRC cells (**A**) Sp1 protein level was detected by Western blot analysis after transfection with Sp1 in SW620 and LoVo cells. (**B**, **C**) MTT and colony formation assays were performed in SW620 and LoVo cells transfected with miR-22, Sp1 or miR-22/Sp1. (**D**, **E**) Transwell migration and invasion assays were performed in SW620 and LoVo cells transfected as above. *P < 0.05, **P < 0.01.

To confirm that miR-22's effect on CRC cells is due to its down- regulation of Sp1, we performed a rescue experiment by introducing pcDNA3.1-Sp1 plasmid without 3′-UTR or empty vector in the presence or absence of ectopic miR-22 expression in CRC cells. As shown in Figure 5A, Sp1 expression was significantly increased by transfecting SW620 and LoVo cells with pcDNA3.1-Sp1 plasmid compared with its expression in the controls. Then, we found that ectopic Sp1 expression promoted the proliferation, colony formation, migration, and invasion of SW620 and LoVo cells (Figure 5B–5E). Furthermore, reintroduction of Sp1 could partly reverse the inhibitory effects induced by miR-22 in CRC cells (Figure 5B–5E). Thus, these results demonstrate that miR-22 suppresses growth and invasion of CRC cells, at least in part, via directly down-regulating Sp1.

### Sp1 suppresses miR-22 expression to promote CRC cell aggressiveness

**Figure 6:**
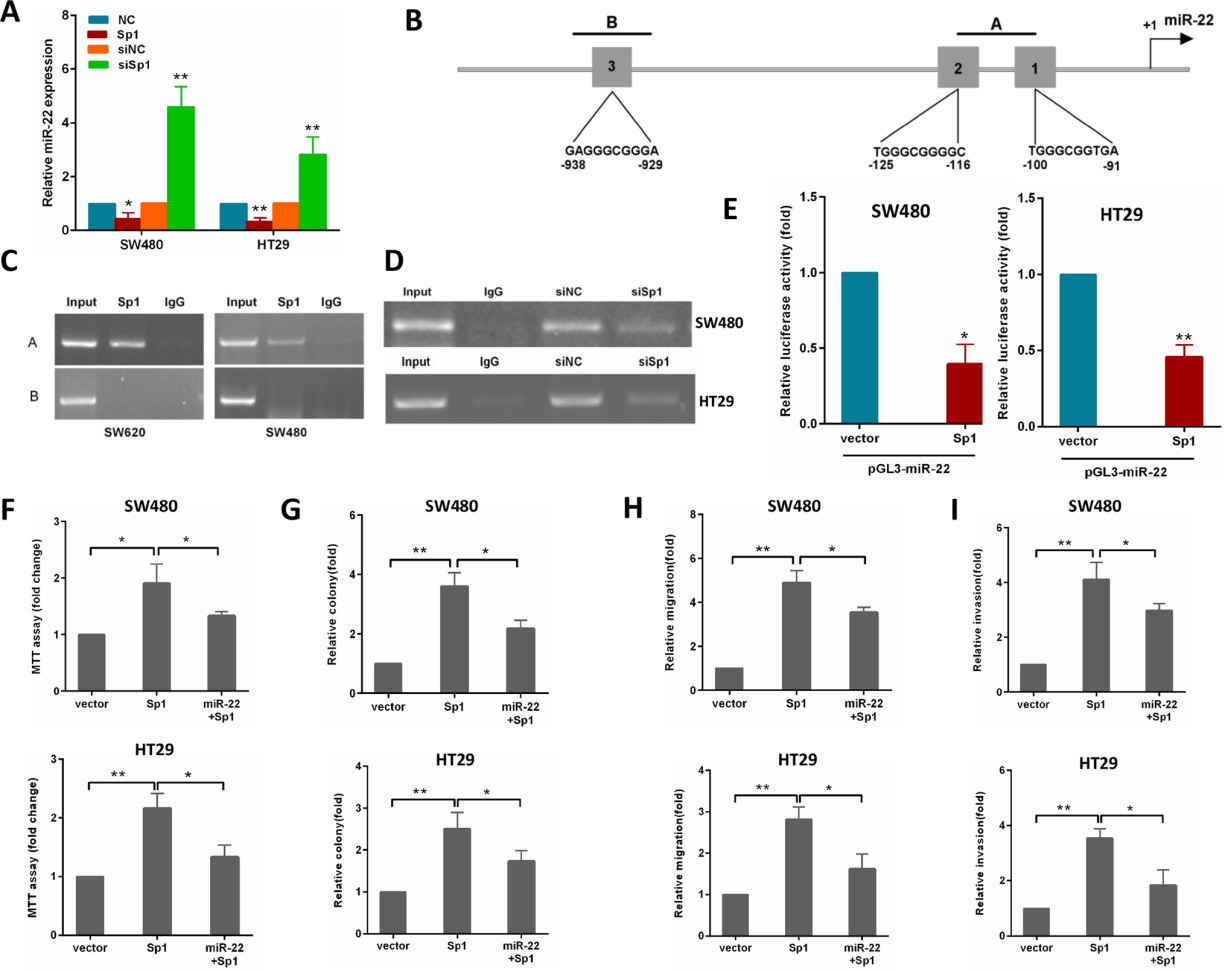
Sp1 binds to the promoter of miR-22 and depends on the miR-22 to promote CRC aggressiveness (**A**) miR-22 levels were analyzed by qRT-PCR in SW480 cells transfected with Sp1 or siSp1. (**B**) schematic diagram indicates the location and sequences of 3 putative Sp1-binding sites on miR-22 promoter region. (**C**) ChIP assays were performed in SW620 and SW480 cells with anti-Sp1 antibody. (**D**) ChIP assay was performed in SW480 and HT29 cells transfected with a control or siSp1. (**E**) Analysis of the luciferase activity of pGL3-miR-22 promoter report plasmid cotransfected with an empty vector or Sp1 plasmid in SW480 and HT29 cells. (**F**, **G**) MTT and colony formation assays were performed in SW480 and HT29 cells transfected with Sp1 alone or in combination with miR-22. (**H**, **I**) Transwell migration and invasion assays were performed in SW480 and HT29 cells transfected as above. *P < 0.05, **P < 0.01.

Considering that Sp1 is a transcriptional factor, we explored the possibility that Sp1 may directly regulate the transcription of the miR-22. To examine this possibility, the gain and loss of Sp1 functions were used to study its influence on miR-22 expression. As shown in Figure 6A, ectopic expression of Sp1 reduced miR-22 level, whereas depletion of Sp1 resulted in an increased miR-22 level in SW480 and HT29 cells.

To address whether Sp1 directly regulates miR-22 expression, we performed ChIP assays in CRC cells. By using TRANSFAC, JASPAR and PROMO online sofwares, we found three potential Sp1-binding sites within the promoters of miR-22 (Figure 6B). In the miR-22 promoter, we generated amplicon A which overlaps site 1 and 2, amplicon B which overlaps site 3 (Figure 6B). The ChIP-PCR showed that only amplicon A were amplifed in SW620 cells (Figure 6C). Similar results were detected in SW480 cells (Figure 6C). Moreover, knocking down Sp1 by siRNA in SW480 and HT29 cells reduced the binding of miR-22 promoter to Sp1 protein (Figure 6D). To provide further evidence that miR-22 is a direct transcriptional target of Sp1, we constructed reporter plasmids containing the miR-22 promoter. A dual-luciferase reporter assay revealed that ectopic expression of Sp1 effectively inhibited miR-22 promoter activity in SW480 and HT29 cells (Figure 6E). These findings indicated that Sp1 protein directly interacts with the miR-22 promoter.

To confirm whether Sp1 promotes growth, migration and invasion of CRC cells through miR-22, we performed a rescue experiment by introducing pcDNA3.1-Sp1 plasmid or empty vector in the presence or absence of ectopic miR-22 expression in SW480 and HT29 cells. The results showed that miR-22 incompletely reversed the Sp1-induced proliferation, colony formation, migration and invasion in CRC cells (Figure 6F–6I).

Collectively, our data indicate that miR-22 forms an autoregulatory loop with Sp1 to regulate CRC cell aggressiveness.

### MiR-22 suppresses PTEN/AKT pathway by targeting Sp1 expression

**Figure 7:**
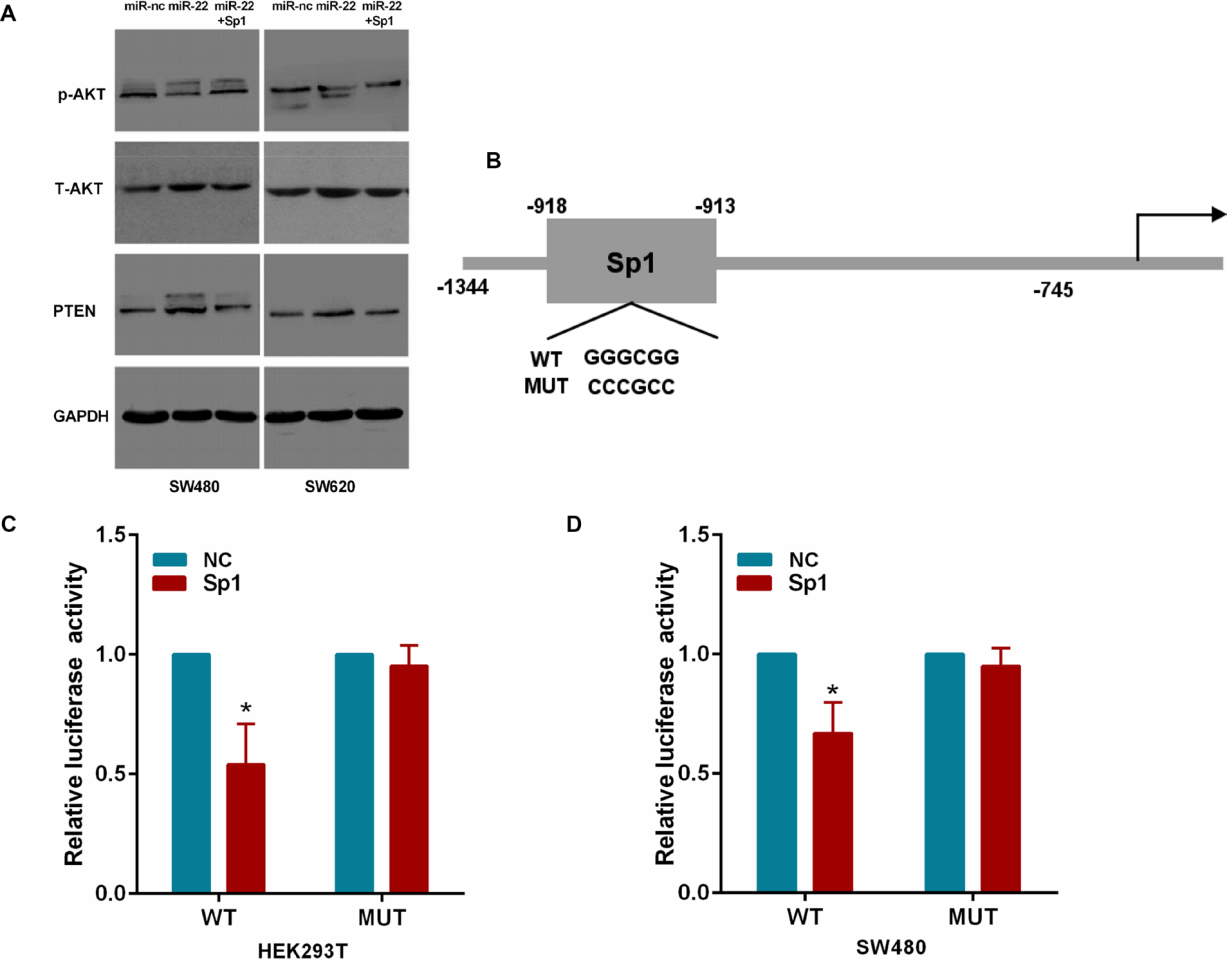
MiR-22 affects PTEN/AKT pathway through Sp1 (**A**) The PTEN, p-AKT and T-AKT protein levels were analyzed by Western blot in SW480 and SW620 cells transfected with miR-22 and Sp1 expression plasmid. (**B**) The Sp1 binding site on the PTEN promoter. The wild-type and mutant Sp1 binding site are shown. (**C**, **D**) Relative luciferase activity in Sp1 or control expressing HEK293T and SW480 cells cotransfected with a wild-type or mutant PTEN promoter. *P < 0.05.

To ascertain that miR-22 regulates PTEN/AKT expression by Sp1 in CRC cells, we cotransfected SW480 and SW620 cells with miR-22 and the Sp1 plasmid. As shown in Figure 7A, we found that miR-22 significantly increased PTEN expression and correspondingly reduced the expression of the phosphorylated AKT (p-AKT) without changing the total level of AKT (T-AKT). However, Sp1 overexpression attenuated the roles of miR-22 in the above effects. Moreover, we transfected HEK293T and SW480 cells with Sp1 plasmid and luciferase reporter containing the wild or mutant type PTEN promoter (Figure 7B). As a result, Sp1 expression significantly decreased the luciferase activity of wild type PTEN promoter reporter, but not that of the mutant reporter (Figure 7C, 7D). Taken together, these results indicate that miR-22/Sp1 network regulates PTEN/AKT pathway in CRC.

## DISCUSSION

In this study, our results showed that miR-22 was significantly down-regulated in liver metastatic tissues than their primary tumors, which was consistent with our previous studies [20]. We further confirmed miR-22 expression was down- regulated in late-stage CRC and was associated with lymph node metastasis and relapse of CRC patients. These results indicate that miR-22 may play an important role in the development and progression of CRC, especially in the processes of metastasis.

In the present study, we demonstrated that miR-22 expression was significantly decreased in CRC cell lines with metastatic capacity compared with those without metastasis. Then, we further performed gain and loss function assays in CRC cells, and found that up-regulation of miR-22 inhibits, while down-regulation of miR-22 promotes proliferation, colony formation, migration and invasion of CRC cells. However, our findings have yielded a contradiction to recent studies on the roles of miR-22 in tumor growth and metastasis [30, 31]. The discrepancies in miR-22's functions in different types of cancer cells may reflect the differences of cellular phenotypic and functional heterogeneity.

In a number of cancers, miRNAs regulated cell proliferation and metastasis by targeting Sp1 [32, 33]. Sp1, a member of the transcription factor family, plays an important role in colorectal cancer development and progression [27, 34]. In this report, we identified Sp1 as a novel, direct target of miR-22 using luciferase reporter assays. This observation was confirmed by the fact that miR-22 overexpression diminished but miR-22 knockdown increased Sp1 mRNA and protein expression in CRC cells. Moreover, ectopic expression of Sp1 significantly compromised the inhibitory effects mediated by miR-22. These results demonstrate for the first time that miR-22 can inhibit CRC cell proliferation, colony formation, migration and invasion by directly targeting its target gene Sp1.

Sp1 has been reported to regulate the expression of its target gene via binding to their promoters. It often works through binding to GC-rich promoter elements with a consensus sequence 5′-(G/T)GGGCG G(G/A) (G/A)(G/T)-3′ [35]. Sp1-induced transcription of protein coding genes has been extensively studied in human cancers [36, 37]. However, the role of Sp1 in the transcription of non–protein coding genes, such as miRNAs, is less explored. Recent studies indicated that the Sp1 transcription factor plays an important role in miRNA expression. For example, Sp1-driven up-regulation of miR-19a decreases RHOB and promotes pancreatic cancer cell proliferation, migration and invasion [38]. Sp1-mediated microRNA-182 expression regulates lung cancer progression [39]. Sp1-mediated microRNA-520d-5p suppresses tumor growth and metastasis in colorectal cancer by targeting CTHRC1 [40]. In our study, Sp1 activation resulted in the down-regulation of miR-22 in CRC cells. Conversely, inhibition of Sp1 strongly increased miR-22 expression levels. Moreover, Sp1 could bind to the promoter of miR-22, suggesting that Sp1 is a direct transcriptional suppressor of miR-22. This is in accordance with a previous result in breast cancer [41]. The present study further conformed that miR-22 could partially inverse Sp1-induced proliferation, colony formation, migration and invasion in CRC cells. These observations provided strong evidences that the suppression of miR-22 by Sp1 activation is important for CRC cell growth and metastasis.

PTEN is a key tumor suppressor gene that antagonizes the PI3K-AKT signaling pathway in cancer cells [42]. The PTEN/PI3K/AKT pathway is highly involved in CRC progression [43, 44]. A previous report demonstrated that Sp1 can inhibit PTEN expression by binding to the PTEN promoter region [45]. For this reason, we wondered whether miR-22 could suppress Sp1 expression to inhibit PTEN/AKT pathway. Here, we found that miR-22 significantly increased PTEN expression and correspondingly reduced the expression of p-AKT. However, this phenomenon could be attenuated by Sp1. Moreover, Sp1 could bind to the promoter of PTEN, which was consistent with a previous study of tongue squamous cell carcinoma [46]. Taken together, miR-22 may inhibit PTEN/AKT pathway by targeting Sp1 expression.

The interaction between miR-22 and Sp1 has been reported in many cancers [19, 41]. However, there are many novel discoveries in the present study. Firstly, we found miR-22 expression was associated with advanced clinical stage and relapse-free survival rate in CRC. This result was not previously reported. Secondly, we provided the first evidence that the miR-22 forms a negative feedback loop with Sp1 in CRC cell growth and metastasis. Thirdly, our study demonstrated for the first time that miR-22/Sp1 network inhibits PTEN/AKT pathway in colorectal cancer. In addition, our results revealed Sp1 inhibits PTEN expression through binding to PTEN promoter, which was not reported in CRC before. Thus, our study contributes novelties to this field.

**Figure 8:**
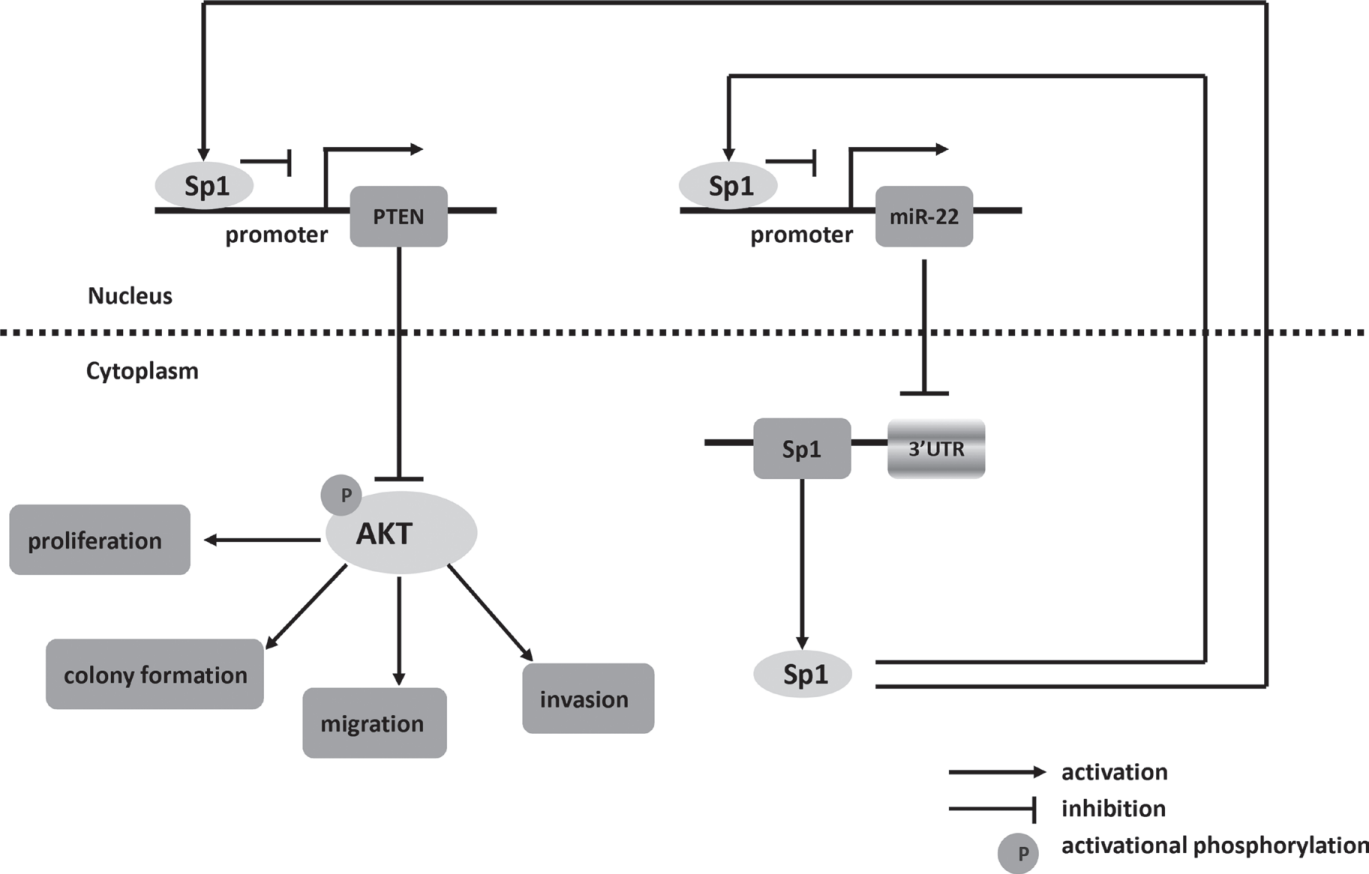
Schematic of pathways involved in the tumor-suppressor role of miR-22 in CRC cells

In summary, we find that miR-22 and Sp1 form a double-negative feedback loop and consequently activation of PTEN, leading to a decline of p-AKT which influences the biological features of cells (Figure 8). Our results reveal that the miR-22/Sp1/PTEN/AKT axis might elucidate the complex molecular mechanisms which regulate progression and metastasis in CRC, and represents a novel strategy for prognostic prediction and the treatment of patients with CRC.

## MATERIALS AND METHODS

### Patients and tissue samples

We enrolled 118 CRC patients who underwent surgery at The Affiliated Hospital of North Sichuan Medical College, from January 2007 to January 2011. The characteristics of the patients are shown in Supplementary Table 1. After collection, all tissue samples were immediately frozen in liquid nitrogen and stored at −80°C until use. None of patients had received preoperative adjuvant therapy. Tumor stage was classified according to the 7th edition of the UICC/AJCC TNM staging system for CRC. The median follow-up period was 66 months (range, 10–82months). Relapse-free survival was defined as the time from surgery to first tumor recurrence (local recurrence and/or distal metastasis). Informed written consent was obtained from each patient, and research protocols were approved by the Medical Ethics Committee of North Sichuan Medical College.

### Cell culture

The human CRC cell lines (SW480, LoVo, HT29, SW620 and Caco-2) and the human embryonic kidney cell line 293T were obtained from the American Type Culture Collection. The CRC cell lines and 293T cells were cultured in DMEM (Invitrogen, Carlsbad, CA, USA) supplemented with 10 % fetal bovine serum (FBS; Invitrogen). All cells were maintained in a humidified incubator at 37°C with 5% CO_2_.

### Oligonucleotides and plasmid transfection

MiR-22 mimics or inhibitor (anti-miR-22) and their negative controls (miR-nc mimics or anti-miR-nc) were obtained from RiboBio (Guangzhou, China). The following sense sequence of siRNA oligonucleotides was used to target the Sp1 transcripts: si-Sp1: 5′-CACAAACACTGCCCACCG-3′ (Invitrogen). Scrambled siRNA was used as a negative control. The coding sequences of Sp1 that was amplified by PCR and subcloned into vector pcDNA 3.1(Invitrogen) using the primers listed in Supplementary Table 2. The empty vector served as a negative control. Transfection was carried out using Lipofectamine 2000 reagent (Invitrogen) according to the manufacturer's instructions.

### Lentiviral transduction and transfection

Lentiviral constructs expressing miR-22 (Lenti-miR microRNA precursor clone collection; System Biosciences, Carlsbad, CA, USA) were packaged using the pPACKH1 lenti-vector Packaging Kit (System Biosciences) in HEK293T cells. The scramble control hairpin pCDH-CMV-MCS-EF1-copGFP was purchased from the same vendor for negative control. The virus particles were harvested 3 days after transfection. SW620 cells were infected with recombinant lentivirus- transducing units supplemented with 8 mg/ml Polybrene (Sigma, St Louis, MO, USA). The stable transfected cells were selected using puromycin and confirmed by qRT-PCR.

### RNA extraction and quantitative real-time PCR

Total RNA, including miRNA, was isolated from tissues or cell lines using TRIzol reagent (Invitrogen) according to manufacturer's instructions. For miRNA expression analysis, reverse transcription was performed using the TaqMan microRNA reverse transcription kit (Applied Biosystems, Foster City, CA, USA) with miR-22 specific primers (Applied Biosystems). Mature miR-22 levels were quantified with TaqMan miRNA assays (Applied Biosystems). For Sp1 mRNA detection, reverse transcription was performed using the PrimeScript RT reagent Kit (Takara, Dalian, China). Quantitative PCR was performed using SYBR Premix Ex Taq (Takara) on the ABI 7500 real-time PCR System (Applied Biosystems). U6 snRNA or β-actin was used as internal control. The primer Sequences are listed in Supplementary Table 2. The relative expression levels were calculated by the equation 2^−ΔΔCT^.

### Cell proliferation analysis

Cell proliferation was measured using the MTT assay. Briefly, the transfected cells were plated in 96-well plates at 5 × 10^3^ per well in a final volume of 100 μl, and 20 μl of 5 mg/ml MTT was added to each well at 24, 48, 72 and 96 h. After incubation at 37°C for 4 h, the MTT solution was removed, and 150 μl dimethyl sulfoxide (DMSO) was added to each well followed by measuring the absorbance at 570 nm on a SpectraMax M5 microplate reader (Molecular Devices, Sunnyvale, CA, USA).

### Colony formation assay

The cells were plated in 6-well plates at 500 per well after transfection. After 2 weeks, the cells were washed twice with PBS, fixed with methanol and stained with 0.5% crystal violet. The number of colonies was counted under a microscope.

### Migration and invasion assays

For the cell migration and invasion assay, cells (1 × 10^5^) in serum-free medium were placed into the upper chamber of a 24-well Transwell Chamber (8 μm pore size, Corning Costar Corporation, Cambridge, MA, USA) uncoated or coated with Matrigel (BD Biosciences, San Jose, CA, USA) after transfection. The chambers were incubated for 48 h with culture medium containing 10% FBS added to the lower chamber. The non-invaded cells were removed with cotton swabs. Cells which had invaded to the lower surface were fixed, stained and counted using an inverted microscope (20×). All experiments were performed in triplicate.

### *In vivo* experiments

Female BALB/C nude mice (4–6 weeks old) were purchased from Shanghai Laboratory Animal Center (Shanghai, China). All animal studies were conducted according to protocols approved by the Committee on the Ethics of Experimental Animal of North Sichuan Medical College. To evaluate the *in vivo* tumorigenic effects, 1 × 10^6^ cells were injected subcutaneously into the flank of nude mice (*n* = 5 per group). Tumor size was measured with calipers to estimate volume every 7 days until day 28 after injection. The mice were sacrificed and tumors were collected 28 days later. Tumor volume (V) was calculated as follows: V = length × width^2^ /2. For tail vein metastasis assay, 2 × 10^6^ cells were injected into the tail vein of nude mice (*n* = 6 per group). After 8 weeks, mice were sacrificed and lungs were removed, paraffin embedded and subjected to pathological examination. The number of tumor colonies was determined by using a dissecting microscope.

### Western blot analysis

Cultured or transfected cells were lysed with RIPA lysis buffer containing protease/phosphatase inhibitor Cocktail (Cell Signaling Technology, Beverly, MA. USA). Proteins were separated via SDS-PAGE and transferred onto PVDF membrane. After blocking, the membrane was probed with primary antibodies against Sp1 (1:500; Santa Cruz Biotechnology, Santa Cruz, CA, USA), PTEN (1:500; Santa Cruz Biotechnology), p-AKT (1:1000, Cell Signaling Technology), total AKT (1:1000, Cell Signaling Technology) and GAPDH (1:500, Santa Cruz Biotechnology) overnight at 4°C, followed by incubation with HRP-conjugated secondary antibody (Santa Cruz Biotechnology). Signals were visualized using ECL regents (Millipore, MA, USA).

### Dual-luciferase reporter assay

To validate whether Sp1 are direct targets of miR-22, wild-type or mutant 3′-UTR of Sp1 were cloned into the psicheck-2 vector (Promega, Madison, WI, USA). HEK293T cells were co-transfected with miR-22 mimics or controls and wild-type or mutant 3′-UTR-luc by using Lipofectamine 2000. To validate the Sp1-binding sites in the miR-22 promoter, the miR-22 promoter region (−1100/+55 bp, as was described previously [47]) was amplified from human genomic DNA to generate miR-22 promoter using specific primers. The PCR product was cloned into the pGL3-basic vector (Promega). SW480 cells were transfected with pGL3-miR-22 along with pcDNA3.1-Sp1 expression vector or an empty vector using Lipofectamine 2000. To validate the Sp1-binding sites in the PTEN promoter, the PTEN promoter region (−1344/−745 bp, as was described previously [48]) was amplified from human genomic DNA to generate wild promoter using specific primers. The mutant type in the putative Sp1-binding site in the wild-type fragment (−918 to -913 wild- type: 5′-GGGCGG-3′, the mutant: 5′-CCCGCC-3′) was also PCR- amplified. The wild-type and mutant reporter constructs along with pcDNA3.1-Sp1 expression vector or an empty vector were cotransfected into SW480 and HEK293T cells using Lipofectamine 2000. After transfection for 48 h, cells were harvested and assayed with Dual- Luciferase Reporter Assay System (Promega) according to the manufacturer's protocols. The primers used in aforementioned construction or mutation are listed in Supplementary Table 2.

### Chromatin immunoprecipitation

ChIP assay was performed using EZ ChIP Assay Kit (Millipore) following the manufacturer's instructions. Briefly, SW480 and SW620 cells (2 × 10^6^) were fixed in 1% formaldehyde to cross-link DNA and proteins. Then, the cells were lysed and chromatin was sheared to an average size of 400 bp DNA fragments. Input DNA was stored for following assay. Remaining sheared DNA was incubated with Anti-Sp1 antibody (Santa Cruz Biotechnology). Normal Rabbit IgG was used as negative control. The samples were then reversed and purified. The immunoprecipitated DNA was amplified by PCR for sequences containing Sp1-binding sites. The primer sequences for PCR analysis are listed in Supplementary Table 2.

### Statistical analysis

For continuous variables, data are expressed as mean ± standard deviation (SD). Statistical significance between groups was analyzed by Student's *t*-test or Mann–Whitney *U* test, while categorical data were studied using chi-square test. The postoperative survival rate was analyzed with Kaplan–Meier method and the survival differences were compared by the log-rank test. Statistical analyses were conducted using IBM SPSS Statistics (Version 19, IBM SPSS, Chicago, IL, USA). Statistical significance was defined as *P* < 0.05.

## SUPPLEMENTARY MATERIALS TABLES



## Abbreviations

CRC: colorectal cancer
miR: microRNAs
Sp1: Specificity protein 1
3′-UTR: 3′- untranslated regions
GAPDH: glyceraldehyde-3-phosphate dehydrogenase
PBS: phosphate-buffered saline
RT-PCR: reverse transcription-polymerase chain reaction
PTEN: phosphatase and tensin homologue
