# Supplementary material for: MicroRNA-22 suppresses the growth, migration and invasion of colorectal cancer cells through a Sp1 negative feedback loop
Source: Oncotarget. 2017 Mar 31;8(22):36266–78. doi: 10.18632/oncotarget.16742 (PMC5482653; doi:10.18632/oncotarget.16742)
Supplement: Supplementary file 1 [file oncotarget-08-36266-s001.pdf]

MicroRNA-22 suppresses the growth, migration and invasion of colorectal cancer cells through a Sp1 negative feedback loop

Supplementary Materials

Supplementary Table 1: Characteristics of 118 patients with colorectal cancer

Variable		No. of patients
Gender	Male : female	71:47
Age (years)	Mean (S.D.)	62.6 (12.5)
Tumor size (cm)	≤ 5 cm: > 5 cm	77:41
Location	Colon : rectum	52:66
Histological type	Well : mod : other	25:58:35
Depth of tumor invasion	pT1:pT2:pT3:pT4	24:33:32:29
Lymph node metastasis	Negative : positive	70:48
Distant metastasis	Negative : positive	110:8
Stage	I:II:III:IV	24:46:40:8

Supplementary Table 2: Primer Sequences used for PCR amplification

**Primers for real-time quantitative RT-PCR**		
Sp1	5′-CCATACCCCTTAACCCCG-3′	5′-GAATTTTCACTAATGTTTCCCACC-3′
β-actin	5′-CCAAGGCCAACCGCGAGAAGATGAC-3′	5′-AGGGTACATGGTGGTGCCGCCAGAC-3′
**Primer Sequences used for PCR amplification of plasmid construction**		
Sp1	5′-ATCGGGTACCATGAGCGACCAAGATCACTCCAT-3′ (*KpnI*)	5′-ATCGCTCGAGTCAGAAGCCATTGCCACTGATAT-3′(*XhoI*)
**Primers for luciferase plasmids construction**		
miR-22 -pGL3	5′-GCTACTCGAGTCCCCCGCTCATCTAGACTC-3′ (*XhoI*)	5′-GCCAAGCTTCGAGGGGGAGCAAATCACTG-3′(*HindIII*)
PTEN -pGL3 wt	5′-GCTACTCGAGCCATCTCAGCTTTCATCATCAGTC-3′ (*XhoI*)	5′-GCCAAGCTTGCTCAACTCTCAAACTTCCATC-3′(*HindIII*)
PTEN -pGL3 mut	5′-CAGGCCGGCCCCGCCTGATGTGGCGGGACTCTTTATGCGC-3′ (*XhoI*)	5′-CCGCCACATCAGGCGGGGCCGGCCTGCCCGCCCCCTCCGC-3′(*HindIII*)
**Primers for gene 3′UTR luciferase plasmid construction**		
Sp1 wt	5′-CACAACTCGAGTGCTGGTTTACCCTCAACCC-3′ (*XhoI*)	5′-CACAACACAAGCGGCCGCAAAGGCAACAGTGGTGTGGA-3′ (*NotI*)
Sp1 mut	5′-CAGAACGTCGTTCTTTTGTCTCCCAGCAGTAG-3′ (*XhoI*)	5′-AAAAGAACGACGTTCTGTTTTGAGGCCAAACAAG-3′ (*Not*I)
**Primers for ChIP assay promoter-specific PCR**		
miR-22(A)	5′-CAATTCCGCCCGGCAAAAG-3′	5′-ACGCACGAGCTGCGAATG-3′
miR-22(B)	5′-GCTACTCGAGTCCCCCGCTCATCTAGACTC-3′	5′-CCAAAGTGCAAAGGGTCAGC-3′

Supplementary Table 3: Multivariate COX proportional hazards regression model analysis for relapse-free survival in CRC patients

Variables	HR	95% CI	*P*
Gender(male/female)	1.002	0.432–2.322	0.996
Age(years) (≤ 60/> 60)	1.140	0.488–2.661	0.763
Tumor size(cm) (> 5/≤ 5)	1.644	0.687–3.935	0.264
Location(colon/rectum)	1.154	0.550–2.421	0.705
Histological type (poor, muc/well, mod)	2.032	0.887–4.656	0.094
Tumor depth (T3, T4/T1, T2)	1.111	0.539–2.289	0.775
Lymph node metastasis (present/absent)	3.469	1.393–8.640	0.008
miR-22 expression (low/high, medium)	2.603	1.063–6.374	0.036

HR: hazard ratio, CI: confidence interval. well: well-differentiated, mod: moderately differentiated, poor: poorly differentiated, muc: mucinous carcinoma.
